# V-band ultra-fast tunable thin-film lithium niobate Fourier-domain mode-locked optoelectronic oscillator

**DOI:** 10.1038/s41377-025-01988-7

**Published:** 2025-12-11

**Authors:** Rui Ma, Zijun Huang, X. Steve Yao, Peng Hao, Wei Ke, Xinlun Cai

**Affiliations:** 1 State Key Laboratory of Optoelectronic Materials and Technologies, School of Electronics and Information Technology, Sun Yat-sen University, Guangzhou, 510275 China; 2 Photonics Information Innovation Center and Hebei Provincial Center for Optical Sensing Innovations, College of Physics Science and Technology, Hebei University, Baoding, 071002 China; 3 NuVison Photonics, Inc., Las Vegas, NV 89109 USA; 4 Hefei National Laboratory, Hefei, 230088 China

**Keywords:** Optics and photonics, Other photonics

## Abstract

We demonstrate the first Fourier-domain mode-locked optoelectronic oscillator (FDML OEO) fabricated on the thin-film lithium niobate (TFLN) platform, deploying an electrically tuned ultra-fast frequency-scanning filter, thanks to the high-speed Pockels effect in TFLN. Record-breaking high radiofrequency oscillations up to 65 GHz are achieved, with a phase noise more than 14 dB less at 50 GHz than that of a high-performance commercial signal source at an offset frequency of 10 kHz away from the carrier. A linearly chirped microwave waveform with an unprecedented scanning bandwidth of 30 GHz, corresponding to an impressive chirp rate of 5.7 GHz/μs and a large time-bandwidth product of 159054, is successfully generated by the FDML OEO. These results validate the feasibility of utilizing TFLN to fabricate integrated FDML OEOs capable of delivering ultra-wide scanning bandwidth at chirp rates and frequencies not attainable with any other approaches to date.

## Introduction

In advanced radar systems, such as frequency-modulated continuous wave radar, linearly chirped microwave waveform (LCMW) signals with a large time-bandwidth product (TBWP) are highly desired for improving range resolution and detection distance^1,2^. Ideally, an LCMW should feature a fast tuning speed at high frequencies to enhance anti-reconnaissance and anti-jamming capabilities^3–8^. Furthermore, LCMW signals are often used as the driving signal for electro-optic frequency combs^9,10^ in repetition rate-modulated frequency comb ranging systems^11^. When the chirp rate of the LCMW increases, the system can reduce the time interval between adjacent detected pulses, thereby enabling higher data acquisition rates. The conventional method of generating LCMW signals is to utilize a voltage-controlled oscillator^12^ or a digital frequency synthesizer^13^. However, the center frequency and bandwidth are limited by the relatively low speed of electronic devices^14^. Photonic methods are potential solutions to overcome this problem, which holds the inherent advantages of high frequency and large bandwidth. In the past few years, numerous approaches have been reported for LCMW generation, such as optical frequency division^15^, wavelength or space to time mapping^16–18^, optical heterodyne detection^19–21^, but the TBWP is typically limited, and the phase noise deteriorates at high frequencies.

A Fourier-domain mode-locked optoelectronic oscillator (FDML OEO)^22–25^ is capable of generating LCMW with low phase noise and large TBWP at high frequencies. Utilizing low-loss long optical fibers or high-Q optical resonators as energy storage elements can ensure low phase noise^26^. However, a long loop would lead to a long oscillation mode buildup time, thus a low frequency-scanning speed. To generate fast frequency tunable microwave signals, it is essential to break the limitation imposed by the time required for mode buildup^27–32^ using the concept of Fourier-domain mode-locking. FDML OEO can be realized by utilizing a fast frequency-scanning filter, which is driven by a periodic signal with a tuning period equal to the round-trip time of oscillating RF signals circulating in the OEO loop. This process will stimulate a large number of longitudinal modes simultaneously with a fixed phase relationship in the Fourier domain, to force a periodically repeated and stable chirped oscillation directly in the loop. Consequently, the output frequency can be tuned at an ultra-fast speed, without the need for mode buildup. Recently, several FDML OEOs have been demonstrated in silicon on insulator (SOI) platform with thermal-optic frequency-scanning components^33,34^. However, the slow thermo-optic effect in silicon results in a small scanning bandwidth for the LCMW signals, thereby leading to a low chirp rate. Moreover, the switching mechanisms on SOI platforms are inherently nonlinear and sensitive to temperature variations, which makes it challenging to develop a high-performance electro-optic modulator (EOM) capable of supporting high-frequency oscillations. On the other hand, thin-film lithium niobate (TFLN)’s ultra-fast linear electro-optic effect^35–37^ is capable of constructing a fast frequency-scanning filter and a high-performance EOM^38–40^.

In this paper, we demonstrate an FDML OEO fabricated on the TFLN platform in which a fast frequency-scanning filter is realized with an electrically tuned TFLN add-drop micro-ring resonator (MRR). Our device features a record ultra-high oscillating frequency up to 65 GHz, covering both Q-band and V-band. The generated LCMW signal has an unprecedented scanning bandwidth of 30 GHz, a large TBWP of 159054, and an impressive chirp rate of 5.7 GHz/μs, while the OEO loop length is chosen to be 1.064 km. We also demonstrated both 2-level frequency shift keying (FSK)^41–43^, and 4-level FSK signal generation, showcasing the rapid switching capability of our device.

## Results

### Principle

**Fig. 1:**
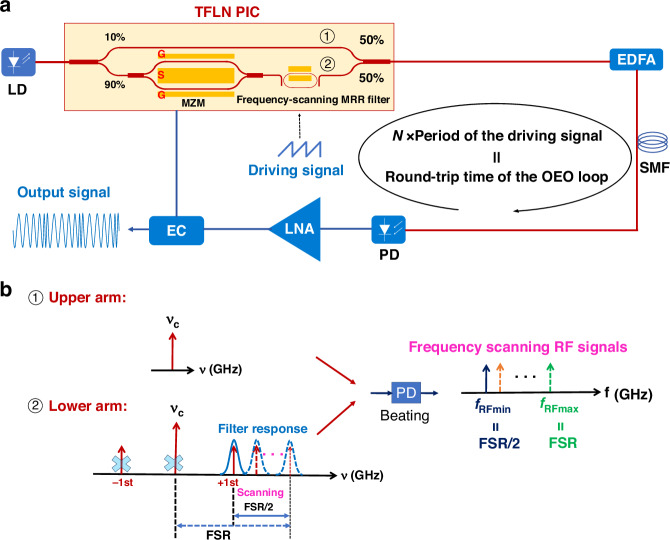
FDML OEO. **a** Schematic; **b** the operation principle

Figure 1a shows the schematic of our FDML OEO, which includes a TFLN photonic integrated chip (PIC) consisting of a Mach-Zehnder modulator (MZM), an electrically tunable add-drop MRR, and two beam splitters with coupling ratios of 10%:90%, and 50%:50%, respectively. The light from a laser is coupled into the PIC and splits into two arms. 90% of the light is coupled to the lower arm containing the MZM and the MRR, which functions as the frequency-scanning filter. The remaining 10% goes to the upper arm before combining and interfering with the light from the lower arm at the 50% beam splitter. The signal output from the PIC is then amplified by an erbium-doped optical fiber amplifier (EDFA) to compensate for the insertion loss of the PIC before going into the OEO loop of a 1 km-long single-mode fiber (SMF) for phase noise reduction and TBWP enhancement. Finally, the optical signal from the SMF will be detected by the PD and amplified by the low-noise amplifier (LNA) before feeding back to the MZM to close the OEO loop. The optoelectronic oscillation will start when the small signal open-loop gain exceeds unity.

To implement the FDML OEO, the MRR acting as the frequency-scanning filter is driven by a periodic signal with a tuning period or its multiple matching the round-trip time of the loop. Each instantaneous frequency component propagates through the cavity and returns to the frequency-scanning filter when its passband is tuned to the same frequency. This ensures that all longitudinal modes are simultaneously active throughout the sweep, enabling the generation of a fast frequency-scanning microwave signal.

Figure 1b depicts the operation principle of our device. The oscillating RF frequency is given by beating the un-modulated optical carrier from the upper arm and the modulation sideband selected by the MRR at the PD. As the MRR passband is swept by an applied periodic driving signal, the frequency of the generated RF signal also sweeps accordingly to give out the LCMW, with a frequency tuning range equal to half of the MRR’s free spectral range (FSR) from FSR/2 to FSR^44^, assuming that the LNA gain is sufficient to cover this frequency range. It is important to note that our device can also be used to generate lower frequency LCMW signals, ranging from 0 to FSR/2, provided the RF LNA operates within this frequency range. Alternatively, by varying the waveform of the driving signal applied to the frequency-scanning filter, our device can generate arbitrary waveform signals such as FSK signals. In contrast to our previous fixed-frequency OEO with a thermally tunable frequency-scanning filter^45^, this device deploys a much faster electrically tunable frequency-scanning filter to generate LCMW signals.

### Frequency-scanning filter performance characterization

**Fig. 2:**
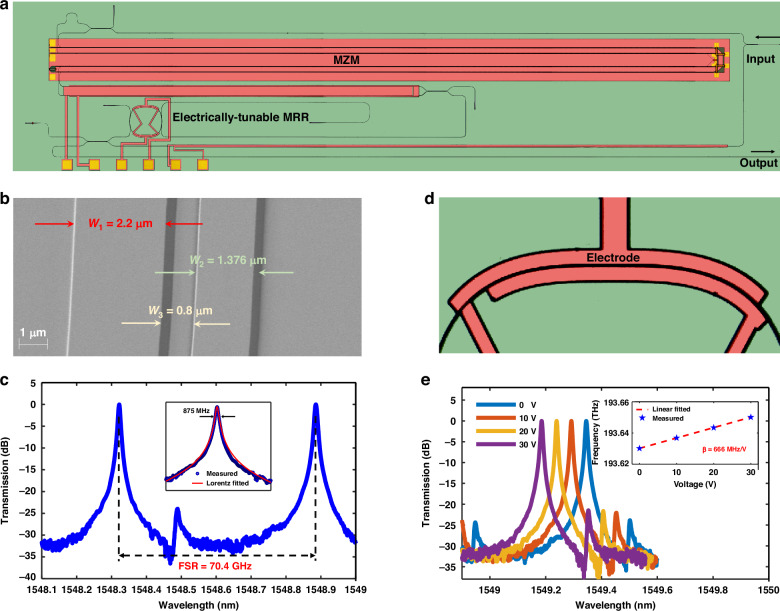
**Experimental results of the TFLN PIC**. **a** The microscope photo of our device; **b** the SEM image of the coupling region of the MRR; **c** the normalized transmission spectrum of the drop port of the MRR (Inset: one of resonances); **d** the microscope image of the tuning gold electrode; **e** the wavelength shift of a resonance of the MRR as a function of the applied DC voltage (Inset: the electrically tunable efficiency)

The PIC for the FDML OEO is designed and fabricated on an X-cut TFLN platform, as depicted in Fig. 2a. The measured half-wave voltage (*V*_π_) and the 3 dB electro-optic (EO) bandwidth of the MZM are 1.3 V and 37.5 GHz, respectively. The detailed performance characterization can be found in the supplementary files.

The fast tunable frequency-scanning filter is the most important element in our device, which is realized by an electrically tunable TFLN add-drop MRR. Figure 2b shows the scanning electron microscope (SEM) image of the coupling region of the MRR. The circumference of the MRR is designed to be 2 mm in order to achieve an FSR of ~70 GHz. For the phase-matching condition, the ring waveguide width (*W*_1_), the bus waveguide width (*W*_2_), the gap (*W*_3_), and the coupling length between them are specially designed to be 2.2 μm, 1.376 μm, 0.8 μm, and 38 μm, respectively. Figure 2c shows the measured 3 dB bandwidth and the FSR of the MRR to be 875 MHz and 70.4 GHz, respectively.

We utilize the Pockels effect of TFLN to achieve a high-speed frequency-scanning filter. Figure 2d shows the microscope image of the gold tuning electrode. The thickness of the gold layer is designed to be 0.9 μm, while the gap between the two gold electrodes is set at 3.2 μm. Figure 2e demonstrates that the resonance peak of MRR can be tuned from 1549.3454 to 1549.1855 nm by applying the voltage from 0 to 30 V, resulting in an electrical tuning efficiency of 0.666 GHz/V. The maximum frequency-scanning speed of the TFLN MRR filter is measured to be 3 MHz in our work by applying sawtooth signals, which can be improved by optimizing the electrode design of the MRR, though ultimately it is limited by the electro-optic effect of TFLN.

### Fixed-frequency OEO

**Fig. 3:**
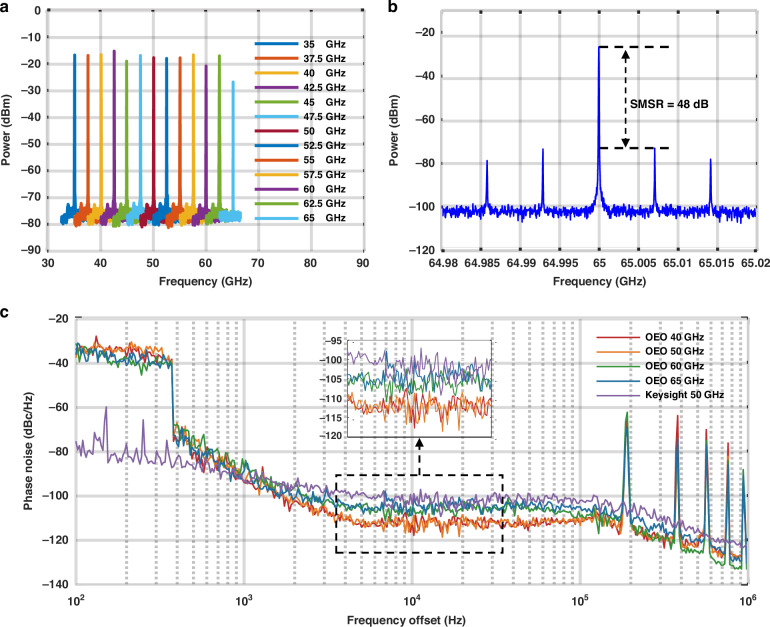
**Experimental results of the fixed-frequency OEO**. **a** The wide range frequency tunability of the fixed-frequency OEO without using a long-length SMF; **b** the RF spectrum of the 65 GHz signal generated by the OEO measured with an RFSA with a frequency span of 40 MHz and a resolution bandwidth (RBW) of 40 kHz; **c** measured phase noise curves of the 40 (red curve), 50 (orange curve), 60 (green curve), and 65 GHz (blue curve) oscillations of the fixed-frequency OEO with a loop length of 1.064 km, as compared with that of a 50 GHz signal from a commercial microwave source (Keysight E8257D)

We first demonstrate the capability of generating different fixed RF oscillations without using a long-length SMF. As shown in Fig. 3a, the oscillating frequency can be adjusted from 35 to 65 GHz by applying a DC voltage in the range of −22.5 V to 22.5 V to the MRR, measured with the RF spectrum analyzer (RFSA) (Agilent Technologies PXA N9030A). This represents the highest oscillation frequency ever reported among on-chip OEOs. Note that PXA N9030A has a maximum frequency range of 50 GHz such that for measuring a frequency above 50 GHz, a mixer (Marki Microwave MM1-1857LS) must be used to downconvert it to a frequency below 50 GHz. It can be seen that the measured oscillation power at 65 GHz is 10 dB lower than that at 35 GHz, which is partially attributed to the increase of more than 8 dB conversion loss from the mixer and 1 dB from the coax cable of the 65 GHz signal as compared to the 35 GHz signal. Figure 3b shows the frequency spectrum of the generated RF signal when the OEO is tuned to 65 GHz. The mode spacing, primarily determined by the total signal path length of all optical and RF components in the OEO loop, is 7 MHz, with a side mode suppression ratio (SMSR) of 48 dB.

Figure 3c shows the phase noise curves of the fixed-frequency OEO utilizing a 1 km-long SMF, measured with PXA N9030A having a phase noise measurement plug-in module. The red, orange, green, and blue curves represent the phase noises of the 40, 50, 60, and 65 GHz signals, respectively, while the purple curve represents the phase noise of a commercial microwave source (Keysight E8257D) as a reference. It can be seen that the phase noises of the OEO at offsets below 1 kHz are higher than those of the commercial microwave source, which is primarily attributed to the fluctuations of the fiber-chip coupling due to the random mechanical vibrations. Nevertheless, at frequency offsets above 2 kHz, the OEO exhibits lower phase noise than the commercial signal source (Keysight E8257D), benefiting from the long loop delay. At a 10 kHz offset, the phase noise for the 40 and 50 GHz oscillations drops to approximately −118 dBc/Hz, representing the lowest phase noise reported of an OEO with a 1.064 km-long loop delay at these frequencies. On the other hand, the measured phase noises of the 60 and 65 GHz oscillations are −109 dBc/Hz at 10 kHz offset, slightly higher than the oscillations below 50 GHz, but still lower than that of the reference signal at 50 GHz. It should be noticed that the measured phase noises of oscillations at 60 GHz and 65 GHz also include the contributions of the reference signal at 40 GHz and 45 GHz required to down convert the signals at 60 GHz and 65 GHz to below 50 GHz to fit into the frequency range of our RF spectrum and phase noise measurement instrument (PXA N9030A).

### LCMW generation

**Fig. 4:**
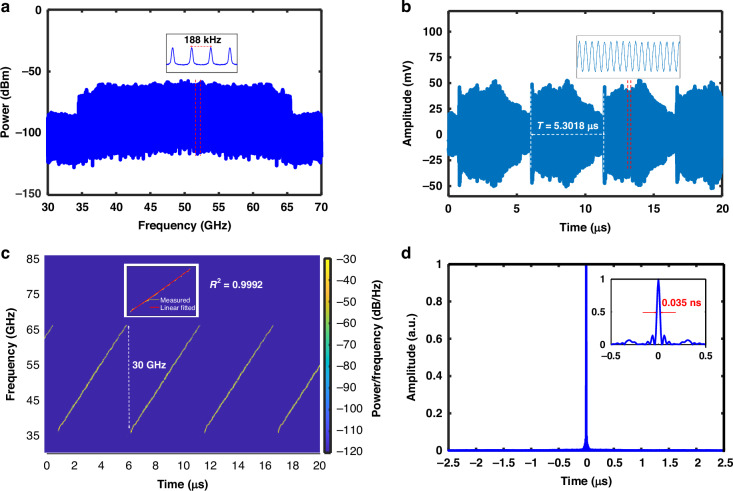
**Experimental results of LCMW signals**. **a** The generated RF spectrum. Inset: a zoomed-in view of the RF spectrum, measured with an RFSA with a span of 700 kHz and a RBW of 9.5 kHz; **b** temporal waveform of the periodically and continuously chirped microwave waveform (Inset: a section of the waveform); **c** real-time frequency distribution (Inset: linear fit with the *R*^2^ value showing the goodness of fit); **d** the compressed pulse by autocorrelation (Inset: zoomed-in view)

Next, we used our FDML OEO to generate LCMW signals. The experimental setup for measuring the generated LCMW signals can be found in the materials and methods. A sawtooth signal with a periodicity of 5.3018 μs, equal to the round-trip time of the OEO loop, is applied to tune the MRR. The scanning bandwidth, the TBWP, the chirp rate, and the pulse compression ratio (PCR) are measured to characterize the LCMW. Figure 4a shows the measured RF spectrum, indicating that all modes are active simultaneously. The inset reveals the mode spacing of 188 kHz, matching the round-trip time. Figure 4b shows the measured microwave waveform in the time domain, with a temporal periodicity of 5.3018 μs, measured using a high-speed real-time oscilloscope (LabMaster 10-36Zi-A) with a sampling rate of 80 GSa/s and a bandwidth of 36 GHz. Figure 4c shows the instantaneous frequency of the generated waveform, obtained by calculating the short-time Fourier transform of the measured microwave waveform. It can be observed that the waveform is periodic, with the instantaneous frequency increasing linearly over each period. The inset shows an *R*-squared value of 0.9992, confirming that the generated LCMW signals have good linearity, which is attributed to the linear response of the frequency-scanning MRR filter. In contrast, for the SOI-based^33^ and silicon nitride-lithium niobate-based^46^ (SiN-LN-based) FDML OEO schemes, the linearity of the LCMW signals is compromised due to the nonlinear thermal-optic effects. The measured scanning bandwidth is 30 GHz, which is unprecedented among FDML OEOs. The maximum instantaneous frequency-scanning range (i.e., scanning bandwidth) is fundamentally determined by the electro-optic effect of the TFLN, which will not be reduced by increasing the frequency-scanning speed of the MRR filter. The corresponding TBWP is calculated to be 159054, with an impressive chirp rate of 5.7 GHz/μs, ~24 times higher than the chirp rate of 239.1 MHz/μs in the SOI FDML OEO^33^. If an LNA and a mixer with a wider bandwidth are available for realizing the theoretical scanning bandwidth of 35 GHz, the TBWP and the chirp rate could reach 185564 and 6.6 GHz/μs, respectively. The chirp rate can be further increased by deploying a shorter optical fiber to reduce the OEO’s round-trip time and consequently reduce the scanning period. Figure 4d shows the autocorrelation of the generated LCMW to evaluate the pulse compression capability. A compressed pulse width of ~0.035 ns is observed, corresponding to a PCR of 151480.

**Fig. 5:**
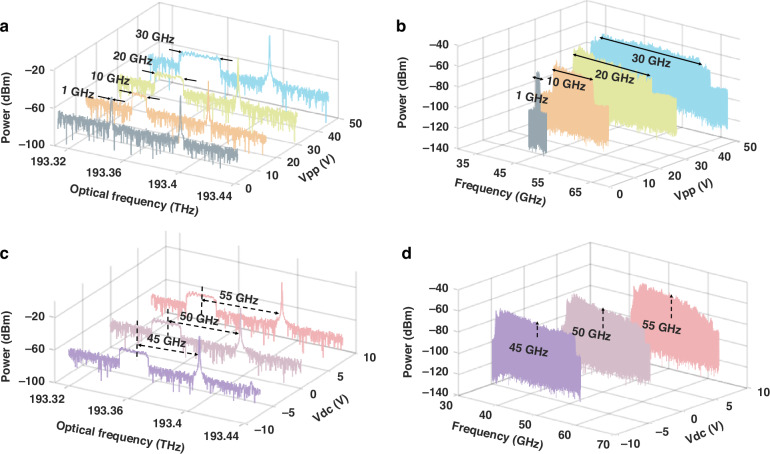
**The reconfigurability of the LCMW signals**. **a** The measured optical spectra when the MRR is driven with sawtooth signals of different Vpp and **b** RF spectra with scanning bandwidths of 1, 10, 20, and 30 GHz, respectively; **c** the measured optical spectra when the MRR is driven with sawtooth signals of different Vdc and **d** RF spectra with scanning center frequencies of 45, 50, and 55 GHz, respectively

Furthermore, we investigate the reconfigurability of the LCMW. The scanning bandwidth and center frequency can be adjusted either by the amplitude (Vpp) or the direct current component (Vdc) of the sawtooth driving signal. Figure 5a, b presents the experimental results of scanning bandwidth tuning by applying sawtooth signals with different Vpp. The measured optical spectra presented in Fig. 5a exhibit identical central wavelengths for the optical carriers, while the modulation sidebands display bandwidths of 1, 10, 20, and 30 GHz, with the Vpp of 1.5 V, 15 V, 30 V, and 45 V, respectively. Figure 5b presents the corresponding measured RF spectra with the bandwidths of 1, 10, 20, and 30 GHz, respectively. Additionally, Fig. 5c, d demonstrates the experimental results of the center frequency tuned by applying sawtooth signals with different Vdc. The measured optical spectra presented in Fig. 5c exhibit an optical carrier with a fixed center frequency (wavelength) and the associated modulation sidebands at 45, 50, and 55 GHz from the carrier, when the Vdc of −7.5 V, 0 V, and 7.5 V is applied, respectively. Figure 5d presents the corresponding measured RF spectra with the center frequencies of 45, 50, and 55 GHz, respectively. The measured temporal waveforms and the corresponding calculated spectrograms of the reconfigurable LCMW signals can be found in the supplementary files.

### FSK generation

**Fig. 6:**
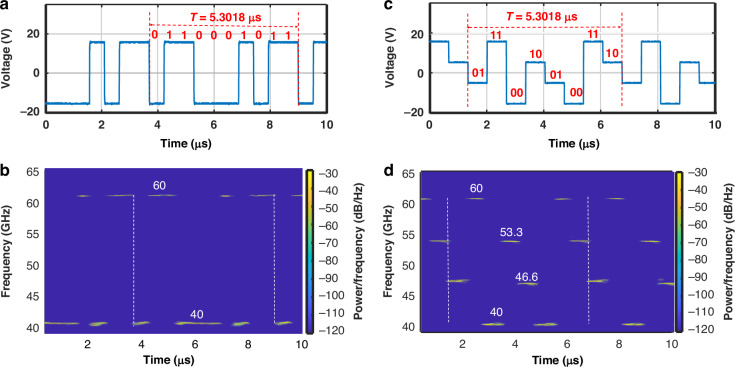
**Experimental results of FSK signals**. **a** A binary driving signal applied to the frequency-scanning filter; **b** the corresponding calculated spectrograms of the generated 2-level FSK signal; **c** a quaternary driving signal applied to the frequency-scanning filter; **d** the corresponding calculated spectrograms of the generated 4-level FSK signal

FSK is a digital modulation technique that represents different binary data by switching between two or more distinct frequencies, which is widely utilized in short-range wireless communication and low-speed data transmission. We demonstrate both 2-level FSK and 4-level FSK signals using the FDML OEO with a loop length of 1.064 km. Figure 6a shows the applied binary waveform with a voltage of −15 V and 15 V, representing a low level “0” and a high level “1”, respectively, which can be labeled as “0110001011” within each period. The corresponding generated spectrogram is shown in Fig. 6b, demonstrating that the 2-level FSK signals can rapidly switch between 40 and 60 GHz, respectively. Figure 6c shows the applied quaternary waveform with voltages of −15 V, −5 V, 5 V, and 15 V, representing “00,” “01,” “10,” and “11,” respectively, which can be labeled as “0111001001001110” within each period. The corresponding generated spectrogram is shown in Fig. 6d, showing that the 4-level FSK signals can rapidly switch between 40, 46.6, 53.3, and 60 GHz, respectively. It is noteworthy that the high-frequency components of the FSK signals exhibit reduced power levels, primarily attributed to the frequency-dependent RF loss in the OEO loop. To obtain FSK signals with identical intensity, it is essential to incorporate RF components with a flat frequency response across the entire scanning bandwidth. The measured optical spectrum, RF spectrum, and the corresponding temporal waveforms of the FSK signals can be found in the supplementary files. The generated FSK signals showcase the rapid switching capability of FDML OEO.

## Discussion

In summary, an FDML OEO on a TFLN platform is experimentally demonstrated. The key element is the fast tunable frequency-scanning filter, realized by an electrically tunable TFLN add-drop MRR. By applying a driving signal to the frequency-scanning filter with a periodicity or its multiples equal to the round-trip time of the loop, the FDML OEO can be realized. Due to the high-speed nature of the Pockels effect in TFLN, the frequency-scanning filter can be tuned rapidly, with the frequency tuning range equal to FSR/2 of the MRR. With a loop length of 1.064 km, our device can generate RF signals at record-high frequencies up to 65 GHz, which is well into V-band and significantly surpasses the maximum frequencies of 17 and 42.5 GHz achieved by SOI-based^33^ and SiN-LN-based^46^ FDML OEOs, respectively, as shown in Table 1. Unfortunately, the 1 km-long fiber is not desirable for a compact and stable OEO. To enhance compactness, the long fiber can be replaced with a fiber coil made of small-diameter fibers or an integrated low-loss SiN delay line through heterogeneous integration. Stability can be further improved by enclosing the fiber in a thermally stabilized box^47^ or by using a phase-locked loop^48^. The phase noises of the generated V-band signals are notably less than those of the Keysight signal source in the offset frequency range from 4 kHz to 100 kHz, particularly more than 10 dB less for the generated signal at 50 GHz. The 188 kHz mode spacing induced by the long fiber is much smaller than the MRR’s 3 dB bandwidth of 875 MHz, potentially broadening the LCMW signal’s instantaneous linewidth due to the possible multimode operation. To reduce the linewidth, mode selectivity can be improved by narrowing the MRR’s bandwidth or using a dual-loop^49^ or parity-time symmetry^50^ structure. However, narrowing the 3 dB bandwidth requires increasing the MRR’s circumference by reducing its FSR, which will limit the maximum frequency tuning range.

**Table 1:** Comparison of our work with the previously reported typical FDML OEOs

FDML OEO	Integrated components	Fiber length (km)	RF frequency (GHz)	Maximum scanning bandwidth (GHz)	TBWP	Maximum chirp rate (GHz/μs)	Phase noise (dBc/Hz at 10 kHz offset)
PSFBG-based^22^	—	4.5	0–18	4	1.67 × 10^5^	0.34	−134.5 (12 GHz)
HNLF-based^27^	—	1	0.4–15	6	2.24 × 10^4^	0.84	—
EBF-based^29^	—	2.2	8.8–9.2	0.4	4.39 × 10^3^	0.036	−127 (9 GHz)
SOI MRR-based^33^	thermally tunable MRR	5.02	11–17	6	1.51 × 10^5^	0.239	—
SiN-LN MRR-based^46^	PM, thermally tunable MRR	0.0468	3–42.5	2	468	13.4	−93 (35 GHz)
TFLN MRR-based (this work)	MZM, electrically tunable MRR	1	35–65	30	1.59 × 10^5^	5.7	−118 (50 GHz)

*PSFBG* phase-shifted fiber Bragg grating, *HNLF* high nonlinear fiber, *EBF* electrical bandpass filter, *PM* phase modulator

The maximum scanning bandwidth of the LCMW signals generated by our FDML OEO is unprecedented at 30 GHz, corresponding to an impressive chirp rate of 5.7 GHz/μs and a large TBWP of 159054, which are superior to the results of the other approaches listed in Table 1. This performance stems from the use of an electrically tunable TFLN MRR filter, which leverages the ultra-fast linear electro-optic effect to significantly surpass the speed limitations of thermally tuned MRRs used in the SOI-based^33^ or SiN-LN-based^46^ FDML OEOs. We also demonstrate the fast reconfigurability of the LCMW, with the center frequencies quickly tunable from 45 to 55 GHz, and the scanning bandwidth rapidly adjustable from 1 to 30 GHz. Finally, we generated 2-level FSK and 4-level FSK signals, showcasing the rapid switching capability of our device. The proposed TFLN FDML OEO holds great advantages, including small size, ultra-high frequency, and ultra-fast tunability, thanks to the superior properties of the TFLN, and may serve to stimulate more research in this exciting field.

## Materials and methods

### TFLN PIC device design and fabrication

The TFLN PIC for realizing the ultra-fast FDML OEO was designed and fabricated in an X-cut TFLN platform with a device layer of 360 nm, as shown in Fig. 2a. First, we patterned and defined all optical waveguides through e-beam lithography and inductively coupled plasma etching processes. Subsequently, a 1-µm-thick SiO_2_ layer was deposited by plasma-enhanced chemical vapor deposition. Next, we used e-beam evaporation to fabricate a NiCr load with a thickness of 0.2 μm and a gold layer with a thickness of 0.9 μm. A lift-off process was followed to fabricate the capacitance-loaded traveling-wave electrodes (CL-TWEs). Here, CL-TWEs with air bridges were used in TFLN modulators to reduce the geometric length of the device while maintaining a low driving voltage. The thicknesses of the silicon oxide above and below the electrode were both 1 μm.

### Experimental setup for measuring the generated LCMW and FSK signals

**Fig. 7:**
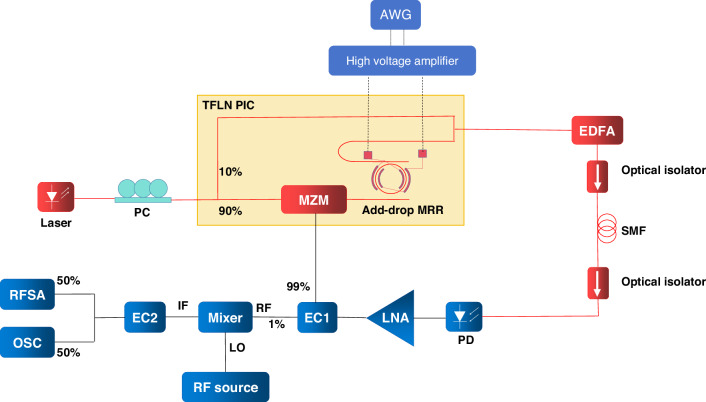
Experimental setup for measuring the generated LCMW and FSK signals

Figure 7 shows the experimental setup for measuring the generated LCMW and FSK signals. A polarization controller (PC) is used to adjust the polarization of light from the laser entering the PIC to be aligned with the TE mode of the PIC. A periodical driving signal provided by an arbitrary waveform generator (AWG, RIGOL DG4202) is amplified by a high voltage amplifier (Falco Systems WMA-300) before tuning the MRR. The first optical isolator is utilized to prevent light from returning to the EDFA (Amonics: AEDFA-PKT-DWDM-15-B-FA). The second optical isolator reduces the impact of Rayleigh scattering from the SMF on the signal. Because PXA N9030 has a maximum frequency range of 50 GHz, for measuring a frequency above 50 GHz, a mixer (Marki Microwave MM1-1857LS) must be used to downconvert it to a frequency below 50 GHz. In addition, the local oscillator (LO) port and the RF port of the mixer both operate in a frequency range of 18–57 GHz, while the intermediate frequency (IF) port operates in a limited frequency range of 0–20 GHz, for measuring a signal with a frequency above 20 GHz, an RF signal from an RF source (Keysight E8257D) is required to down convert the signal to below 20 GHz. 1% of an electrical coupler (EC1) output signal is directed into the mixer to mix with the LO to produce an IF signal, which is then split by EC2. 50% of the EC2 output signal is fed into the oscilloscope (LabMaster 10-36Zi-A) for time-domain measurement, while the remaining 50% is sent to the RFSA for spectral analysis.

## Supplementary information

V-band ultra-fast tunable thin-film lithium niobate Fourier-domain mode-locked optoelectronic oscillator

## Data Availability

The data that support the plots within this paper and other findings of this study are available from the corresponding authors upon reasonable request.
