# Supplementary material for: V-band ultra-fast tunable thin-film lithium niobate Fourier-domain mode-locked optoelectronic oscillator
Source: Light Sci Appl. 2025 Dec 11;14:398. doi: 10.1038/s41377-025-01988-7 (PMC12698762; doi:10.1038/s41377-025-01988-7)
Supplement: Supplementary file 1 — V-band ultra-fast tunable thin-film lithium niobate Fourier-domain mode-locked optoelectronic oscillator [file 41377_2025_1988_MOESM1_ESM.docx]

**Supplementary material**

**V-band ultra-fast tunable thin film lithium niobate Fourier domain mode-locked optoelectronic oscillator**

*Rui Ma ^1,#^, Zijun Huang ^1,#^, X. Steve Yao^2,3,*^, Peng Hao^2,*^, Wei Ke^1^, Xinlun Cai^1,4,*^*

^1^State Key Laboratory of Optoelectronic Materials and Technologies, School of Electronics and Information Technology, Sun Yat-sen University, Guangzhou 510275, China

^2^Photonics Information Innovation Center and Hebei Provincial Center for Optical Sensing, College of Physics Science and Technology, Hebei University, Baoding 071002, China

^3^NuVison Photonics, Inc, Las Vegas, NV 89109, USA

^4^Hefei National Laboratory, Hefei 230088, China

*Corresponding author: [syao@ieee.org](mailto:syao@ieee.org), [haopeng@hbu.edu.cn](mailto:haopeng@hbu.edu.cn), [caixlun5@mail.sysu.edu.cn](mailto:caixlun5@mail.sysu.edu.cn)

*#These authors contributed equally to this work.*

**Section 1. Expression of the LCMW generated with our FDML OEO**

The oscillation RF frequency ($f_{RF}$) is given by beating the un-modulated optical carrier ($f_{0}$) from the upper arm of the MZI and the modulation sideband selected by the MRR filter ($f_{MR}$) at the PD, which can be expressed as

|  | $f_{RF}=f_{MR}-f_{0}=\frac{mc}{nL}-f_{0}$ | (1) |
| --- | --- | --- |

where $m$ is a positive integer, $c$ is the speed of light in vacuum, $n$ and $L$ are the refractive index and the circumference of the TFLN MRR, respectively.

Due to the linear electro-optic effect in the TFLN, the voltage ($V$) applied to the electrically-tunable MRR induces a change in the refractive index ($n$) of the waveguide, such that

|  | $\Delta f_{MR}(t)=-\frac{\Delta n(t)}{n}f_{MR}=\frac{n^{2}\gamma_{33}f_{MR}V(t)}{2d}=\beta V(t)$ | (2) |
| --- | --- | --- |

where$\Delta n(t)=-\frac{n^{3}\gamma_{33}V(t)}{2d}$is refractive index change induced by the applied voltage due to the Pockels effect,$\gamma_{33}$ is the electro-optic coefficient of the TFLN,$d$is the distance between the electrodes located at the two sides of the ring waveguide, and $\beta=\frac{n^{2}\gamma_{33}f_{MR}}{2d}$.

To generate LCMW signals, a sawtooth signal with a periodicity or multiple periodicities equaling to the cavity round-trip time of the OEO loop is applied to tune the MRR, such that

|  | $\Delta f_{MR}=\beta V_{PP}(\frac{t}{T}-\frac{1}{2})$ | (3) |
| --- | --- | --- |

In Eq. (3), $V(t)=V_{PP}(\frac{t}{T}-\frac{1}{2})$ represents the sawtooth wave signal in the first period,$V_{pp}$and T are the peak-to-peak voltage and the periodicity of the sawtooth signal, respectively.

Consequently, the signal output from the PIC can be expressed as

|  | $E_{FDML}\propto E_{0}\left\{\sqrt{b}\cos(2\pi f_{0}t)-\sqrt{a}\sin(\frac{\pi V_{B}}{2V_{\pi}})J_{1}(\frac{\pi V_{0}}{2V_{\pi}})\sin\left\{2\pi\left[f_{MR}+\beta V_{PP}(\frac{t}{T}-\frac{1}{2})\right]t\right\}\right\}$ | (4) |
| --- | --- | --- |

where the first term represents the unmodulated optical carrier from the upper arm, the second term represents one of the modulation sidebands selected by the MRR, $E_{0}$ is the amplitude of the input optical field, $V_{0}$ is the amplitude of the input electrical signal,$a$and b are the loss factors in the upper and lower arms, respectively, $V_{B}$ and $V_{\pi}$ are the bias voltage and half-wave voltage of the MZM, respectively, and $J_{1}$is the first-order Bessel function of the first kind.

The signal output from the PIC is then amplified by an EDFA to compensate for the insertion loss before beating in the PD to produce a beat signal, which is further amplified by a LNA with a voltage gain of $G_{A}$ before feeding back into the MZM to close the OEO loop. The generated LCMW signal can be written as^1,2^

|  | $V_{FDML}\propto RPG_{A}\rho\sqrt{a}\sqrt{b}\sin(\frac{\pi V_{B}}{2V_{\pi}})J_{1}\left(\frac{\pi V_{0}}{2V_{\pi}}\right)\sin\left\{2\pi\left[f_{0}-f_{MR}-\beta V_{PP}(\frac{t}{T}-\frac{1}{2})\right]t\right\}$ | (5) |
| --- | --- | --- |

where$R$, $P$ and $\rho$ are the load impedance, input optical power and responsivity of the PD, respectively. Eq. (5) indicates that the frequency of the generated signals exhibits a linear chirp over time.

**Section 2. TFLN MZM performance characterization**

**Fig.S1 TFLN MZM performance characterization.** a. The measured normalized optical transmission spectrum as a function of the applied DC voltage. (Inset: The transmission spectrum of MZM); b. The measured EO bandwidth (S_21_ parameter) of MZM with a total modulation length of 2.4 cm.

The capacitance-loaded traveling-wave electrodes (CL-TWEs) with air bridges are deployed in the TFLN MZM. This configuration features a total modulation length of 2.4 cm, which effectively reduces the geometric length of the device while ensuring a low driving voltage. Fig. S1a shows that the measured extinction ratio (ER) and V_π_ are 30 dB and 1.3 V, respectively, while Fig. S1b shows that the MZM has a 3dB electro-optic (EO) bandwidth of 37.5 GHz.

**Section 3. The measured temporal waveforms and** **spectrograms of the generated reconfigurable LCMW signals**

**Fig.S2 Scanning bandwidth of the LCMW signals.** a-i, b-i, c-i and d-i showing the temporal waveforms with scanning bandwidths of 1, 10, 20, and 30 GHz, respectively; a-ii, b-ii, c-ii and d-ii showing the corresponding calculated spectrograms.

Fig.S2 shows the measured temporal waveforms and the corresponding calculated spectrograms for the scanning bandwidths of the generated LCMW signals. Figs. S2 a-i, b-i, c-i and d-i display the measured temporal waveforms with scanning bandwidths of 1, 10, 20 and 30 GHz, respectively. For an LCMW signal with a scanning bandwidth of 1 GHz, the temporal waveform amplitude remains fairly constant over one period. In contrast, the temporal waveforms at scanning bandwidths of 10, 20 and 30 GHz exhibit significant amplitude variations over one period. This is due to the frequency-dependence of the modulation, as shown in Fig. S1b, which diminishes the amplitude of higher-frequency components. To effectively mitigate amplitude variations, RF components with a consistent frequency response across the LCMW scanning range can be used. Note that PXA N9030 has a maximum frequency range of 50 GHz and the oscilloscope (LabMaster 10-36Zi-A) has a maximum bandwidth of 36 GHz. In order to measure a frequency above 36 GHz, a 40 GHz signal from a RF source (Keysight E8257D) is fed into the mixer (Marki Microwave MM1-1857LS) to down convert the signal to a frequency below 36 GHz. Figs. S2 a-ii, b-ii, c-ii shows the corresponding spectrogram for the scanning bandwidths of 1 (49.5-50.5 GHz), 10 (45-55 GHz), and 20 GHz (40-60 GHz), respectively. However, since Marki Microwave MM1-1857LS’s IF port operates at a limited frequency range from 0 to 20 GHz, for measuring a scanning bandwidth of 30 GHz, a RF signal of 50 GHz from the RF source (Keysight E8257D) is required to mix with the oscillation signals from 35 to 65 GHz to down convert them to below 20 GHz. The IF output frequency of the mixer first decreases from 15 GHz to 0 GHz and subsequently increases back to 15 GHz in one LCMW period, with the results shown in Figs. S2d-i and S2d-ii.

Figs. S3 a-i, b-i, and c-i are the measured temporal waveforms with the center frequencies of 45, 50, and 55 GHz, respectively, while Figs. S3 a-ii, b-ii, and c-ii display the corresponding calculated spectrograms.

**Fig.S3 Center frequencies of the LCMW signals**. a-i, b-i, and c-i showing the temporal waveforms with the center frequencies of 45, 50 and 55 GHz, respectively; a-ii, b-ii, and c-ii showing the corresponding calculated spectrograms.

**Section 4. Experimental results of the generated FSK signals**

Figs.S4a-S4h are the amplified driving signals, the measured optical and RF spectra, and the corresponding generated temporal waveforms of the 2-level FSK and 4-level FSK signals. Again, because PXA N9030 has a maximum frequency range of 50 GHz and the oscilloscope (LabMaster 10-36Zi-A) has a maximum bandwidth of 36 GHz, for measuring a frequency above 36 GHz, a 39 GHz signal from the RF source (Keysight E8257D) is used to down convert the signals to a frequency below 36 GHz. The slight distortions of the generated temporal waveforms shown in Figs. S4d and S4h are introduced by the driving signals, which are amplified by the high voltage amplifier (Falco Systems WMA-300), as shown in Figs. S4a and S4e. As shown in the measured RF spectrum in Figs. S4c and S4g, the signal power at 60 GHz is lower than that at 40 GHz, which is partially attributed to the higher conversion loss of the mixer (approximately 8 dB) at 60 GHz, as compared to that at 40 GHz.

**Fig.S4 2-level FSK signal generation:** a. The binary driving signal after amplification by the high voltage amplifier (Falco Systems WMA-300); b. The measured optical spectrum including the optical carrier and two hopping modulation sidebands; c. The measured RF spectrum showing the generated RF signals at frequencies of 40 GHz and 60 GHz; d. The corresponding measured temporal waveforms. **4-level FSK signal generation:** e. The quaternary driving signal after amplification by the high voltage amplifier (Falco Systems WMA-300); f. The measured optical spectrum including an optical carrier and four hopping sidebands; g. The measured RF spectrum showing the generated RF signals at frequencies of 40, 46.6, 53.3 and 60 GHz; h. The corresponding measured temporal waveforms.

**Supplementary References**

1. Li, W., Yao, J. A Wideband Frequency Tunable Optoelectronic Oscillator Incorporating a Tunable Microwave Photonic Filter Based on Phase-Modulation to Intensity-Modulation Conversion Using a Phase-Shifted Fiber Bragg Grating. *IEEE Trans. Microw. Theory Tech.* **60,** 1735–1742 (2012).
2. Qiu, H. et al. A Continuously Tunable Sub-Gigahertz Microwave Photonic Bandpass Filter Based on an Ultra-High-Q Silicon Microring Resonator. *J. Light. Technol.* **36**, 4312-4318 (2018).
